# Genetic and Epigenetic Drivers of Wilms Tumor Predisposition in Russian Pediatric Patients: A Multicenter Study

**DOI:** 10.3390/ijms27094066

**Published:** 2026-05-01

**Authors:** Vera Semenova, Garik Sagoyan, Elena Zhukovskaya, Valentina Kozlova, Nina Gegelia, Anna Mitrofanova, Amina Suleymanova, Alexander Druy, Ekaterina Zelenova, Vladislav Pavlov, Marina Rubanskay, Alexander Karelin, Svetlana Varfolomeeva, Tatiana Nasedkina

**Affiliations:** 1 Engelhardt Institute of Molecular Biology, Russian Academy of Sciences, 119991 Moscow, Russia; sulpiridum@yandex.ru (V.S.); zelenovayeye@gmail.com (E.Z.); vladislav1pavlov@gmail.com (V.P.); 2 N.N. Blokhin Russian Cancer Research Center, Ministry of Health of the Russian Federation, 115478 Moscow, Russia; sagoyan-garik@mail.ru (G.S.); valentina-mk2011@yandex.ru (V.K.); aminasuleymanova313@gmail.com (A.S.); marishvecova@yandex.ru (M.R.); s.varfolomeeva@ronc.ru (S.V.); 3 Dmitry Rogachev National Medical Research Center of Pediatric Hematology, Oncology and Immunology, 117198 Moscow, Russia; elena_zhukovskay@mail.ru (E.Z.); gegeliya.nina@fccho-moscow.ru (N.G.); ms.anna.mitrofanova@yandex.ru (A.M.); dr-drui@yandex.ru (A.D.); alexandr.karelin@gmail.com (A.K.)

**Keywords:** Wilms tumor, predisposition, epigenetics, genetic testing, mutations, *WT1* gene, *TRIM28* gene, Beckwith–Wiedemann syndrome

## Abstract

Wilms tumor (WT), the most common kidney neoplasm in children, is closely associated with hereditary factors. This study included 134 WT patients (62 males, median age of 7 years, age at diagnosis of 24.9 months) with unilateral (*n* = 90, 67%) or bilateral WT (*n* = 44, 33%). Genetic testing was performed using targeted sequencing of 415 genes and multiplex ligation–dependent probe amplification (MLPA). Twenty-five mutations in eight genes were found in 17% (*n* = 23) of patients: *WT1* (*n* = 10), *TRIM28* (*n* = 4), *REST* (*n* = 3), *CHEK2* (*n* = 3), *BRCA2* (*n* = 2), *NF1* (*n* = 1), *RAD50* (*n* = 1), and *CDC73* (*n* = 1). Large deletions of the 11p13 region were revealed in 6% (*n* = 5) of patients. The 11p15 locus methylation was studied in blood, tumor, and healthy kidney tissue of nine patients suspected of Beckwith–Wiedemann syndrome (BWS) using methylation-sensitive MLPA (MS–MLPA). BWS was diagnosed in 3% (*n* = 4) of cases (one patient had mosaic disease). Thus, genetic and epigenetic aberrations were identified in 32 WT patients (24%). These patients had a higher frequency of bilateral WT and a higher rate of abnormalities compared to patients without aberrations (56% vs. 25%, *p* = 0.002; and 86% vs. 25%, *p* < 0.0001, respectively). The detection of WT hereditary predisposing factors is crucial for treatment strategies and long-term patient surveillance.

## 1. Introduction

Wilms tumor (WT, nephroblastoma) is an embryonic tumor and the most common childhood renal malignancy, accounting for up to 85% of all pediatric kidney tumors and ranking as the fifth most common cancer in children overall. It is associated with abnormal development of renal tissue and impaired mesenchymal–epithelial transition [1,2]. In two different age groups (0–14 years and 0–17 years), WT accounts for 5.2% and 4.2% of all malignant neoplasms, respectively [3]. Wilms tumor is characterized by early manifestation: in 60% of cases, it is detected between the ages of 1 and 4 (average age of 3 years), and, in 15% of cases, in the first year of life. The incidence of WT in girls is slightly higher than that in boys [3]. In about 5% of cases, bilateral lesions are observed, and in 7%, multiple lesions in one kidney are detected [4].

Depending on study designs and definitions, the authors of previously published reports have identified germline genetic and epigenetic predisposition factors in 5–20% of children with WT [5,6,7]. Most often, mutations occur de novo, and familial forms are observed in only 2% of cases. The most well-known syndromes associated with Wilms tumor are Beckwith–Wiedemann syndrome (BWS), WAGR syndrome (Wilms tumor, aniridia, genitourinary abnormalities, and range of developmental delays), *WT1*-associated syndromes (Denis–Drasch syndrome, Fraser syndrome), Perlman syndrome (*DIS3L2*, recessive) and Simpson–Golabi–Behmel syndrome (*GPC3* and *GPC4*, X-linked recessive) [8,9]. Other syndromes include Fanconi anemia (*BRCA2* or *PALB2*, recessive), Malibray nanism (*TRIM37*, recessive), *PIK3CA*-associated syndromes (somatic), Bohring–Opitz syndrome (*ASXL1*), and osteopathia striata with cranial sclerosis (OSCS) (*AMER1*, X-linked dominant) [9,10,11,12,13,14]. Wilms tumor has also been reported in patients with Bloom syndrome (*BLM*), DICER1 syndrome (*DICER1*), Li–Fraumeni syndrome (*TP53*), and neurofibromatosis type 1 (*NF1*), although the overall estimated risk in these syndromes is considered to be rather low [15,16,17].

Through next-generation sequencing (NGS), the new genes *REST*, *TRIM28*, *CTR9*, *CHEK1*, *EP300*, *ARID1A*, and *NYNRYN* have been identified with germline mutations related to WT [18,19]. Candidate genes have also been discovered in the second Wilms tumor locus, WT2, and two familial Wilms tumor loci, FWT1 and FWT2, have been identified [20]. Thus, the spectrum of genetic changes in hereditary WT syndromes is extremely broad and includes point mutations in heterozygote (*WT1*, *TRIM28*, *REST*, etc.) or homozygote/biallelic states (*DIS3L2*, *BRCA2*, *PALB2*, etc.), large chromosomal rearrangements in the *WT1* gene (WAGR syndrome), as well as methylation pattern abnormalities of the 11p15 chromosome region (BWS). Our understanding of Wilms tumor predisposition continues to evolve, and new genes may potentially be discovered; for this reason, patients with WT require comprehensive genetic testing.

Current treatment regimens include a combination of chemotherapy, surgery and, in some cases, radiotherapy, achieving survival rates of 90% in developed countries [2]. However, advanced-stage WT is still associated with significant morbidity and mortality, making it important to monitor WT in children with various cancer predisposition syndromes (CPSs), *WT1*-associated syndromes, BWS or BWS spectrum (BWSp) to detect tumors at the earliest possible stages [9]. Diagnosis of small tumors enables nephron-sparing surgery, which can also be performed in children with a genetic predisposition, as suggested in the SIOP-RTSG 2016 UMBRELLA protocol [21,22]. Surveillance of Wilms tumor is recommended in familial cases, which are defined as 2 patients with WT in the same family [9].

It should be noted that CPSs are associated with an increased risk of developing bilateral WT [4]. In children with nephroblastomatosis or unilateral WT who have been diagnosed with a CPS, surveillance of the kidneys or remaining kidney is highly warranted. In addition, a number of CPSs significantly increase the risk of developing second tumors during a person’s lifetime; for example, in Li–Fraumeni syndrome, the risk is about 50% [23], while children with BWS are at risk of developing WT and hepatoblastoma [24].

The overall surveillance protocol for children with hereditary predisposition to WT involves ultrasound renal or full abdominal examinations every 3 months until the age of 7 years, depending on the syndrome [9]. If there are structural changes in the kidney, imaging techniques such as magnetic resonance imaging and computed tomography are indicated [4,9]. The use of additional screening measures depends on the precise molecular diagnosis, which is particularly necessary in patients with bilateral WT or malformations. Thus, CPSs associated with WT require timely diagnosis in order to select the optimal algorithm for screening and prevention of secondary tumors. In addition, conducting studies on the molecular mechanisms of hereditary WT development paves the way to search for new therapeutic options.

In this study, we summarized the experience of two large clinical centers in the field of molecular genetic testing and management of patients with Wilms tumor. We identified mutations in both the most frequently damaged genes (*WT1*) and less studied genes (*TRIM28*, *REST*, *CDC73*), discovered new pathogenic genetic variants, analyzed epigenetic aberrations associated with WT, and investigated genotype–phenotype correlations in order to better assess the impact of inheritance on the development of the disease.

## 2. Results

### 2.1. Study Population

The study enrolled 134 patients diagnosed with Wilms tumor during the 6-year observation period from March 2019 to February 2025. The median age at the time of the last observation was 7 years (range 2–20 years), the median age at manifestation was 24.9 months (range 0–147 months), and the sample was predominantly female (54%). The patient characteristics are presented in Table 1.

Most patients were diagnosed with unilateral WT—90/134 (67%), while bilateral kidney involvement was found in 44/134 (33%) patients. A total of 178 affected kidneys were identified in 134 patients (90 tumors in cases of unilateral WT and 88 tumors in cases of bilateral WT), with histological data available for 166 of them. Among the histological subtypes, the stromal (18.6%), regressive (18.6%), mixed (15.6%), epithelial (15%), blastemal (9.0%), and nephroblastomatosis (10.8%) subtypes predominated. Nephroblastomatosis (NBM) is a term used to describe kidneys exhibiting multiple or diffuse nephrogenic rests, putative precursors of WT. Rare histological subtypes included the non-anaplastic subtype (5.4%), complete necrosis (3.0%), tumor with diffuse anaplasia (2.4%), focal anaplasia (0.6%) and fetal rhabdomyomatous nephroblastoma (0.6%). In cases of bilateral WT, various histological subtypes of tumors were found in different kidneys in 15 of 44 patients (34%). In 2.9% of cases (*n* = 4), primary multiple tumors were diagnosed, and in all these cases, the first tumor was WT. The second tumors were retroperitoneal schwannoma, cerebellar glioma, distal small intestine carcinoid, juvenile colon polyps, and osteosarcoma.

Of the 134 patients, five were lost to follow-up, and seven patients were dead; thus, 122/129 (95%) patients were alive after the treatment. Recurrent WT developed in 6/129 (4.7%) patients.

### 2.2. Genetic and Epigenetic Testing

We directed this study to search for genetic and epigenetic predisposition factors in pediatric patients with WT and to characterize the spectrum of aberrations; therefore, attention was primarily focused on patients with bilateral tumors and an abnormal phenotype, as well as on early age of WT onset.

**Figure 1:**
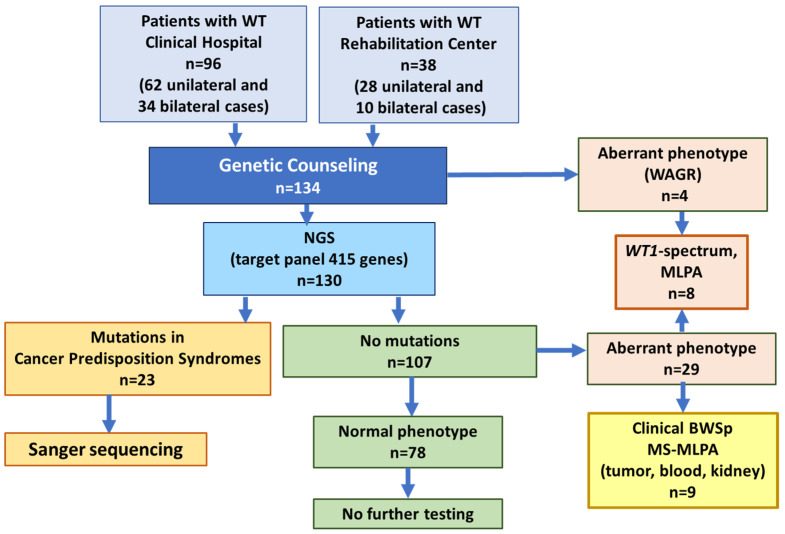
The design of the study.

Through a mixed cohort study, we combined retrospective and prospective data and clinical material collected from 134 patients with WT. All patients were counseled by clinician geneticists, and to search for germline events, DNA isolated from peripheral blood samples was used. In the first stage, 130 patients underwent NGS, while four patients with clinical manifestations of WAGR syndrome or an unusual phenotype were immediately sent to MLPA. In cases where germline mutations in the CPS genes were identified (*n* = 23), the results were confirmed by Sanger sequencing. For patients without mutations who had a normal phenotype (*n* = 78), further testing was not performed. Patients without mutations who were found to have malformations characteristic of *WT1* aberrations were referred for MLPA analysis. Nine patients were found to have clinical features of BWS/BWSp, and their blood, tumor, and intact kidney samples were analyzed by MS-MLPA to distinguish between the classic and mosaic variants of the syndrome (Figure 1).

**Figure 2:**
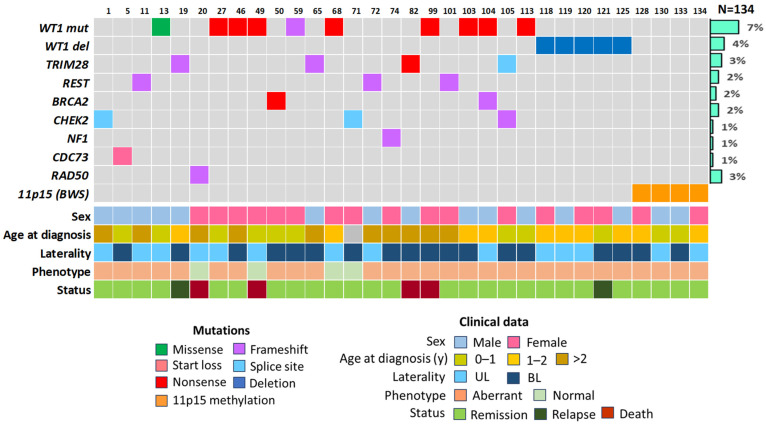
Genetic and epigenetic aberrations in patients with Wilms tumor. UL—unilateral Wilms tumor, BL—bilateral Wilms tumor, mut—mutation, del—deletion.

In total, genetic and epigenetic aberrations in germline DNA were identified in 32 patients with Wilms tumor (24%). When patients were examined using NGS, 23 patients (17%) were found to have 25 heterozygous pathogenic or likely pathogenic genetic variants in eight different genes: *WT1* (*n* = 10), *TRIM28* (*n* = 4), *REST* (*n* = 3), *BRCA2* (*n* = 2), *CHEK2* (*n* = 3), *NF1* (*n* = 1), *RAD50* (*n* = 1), *CDC73* (*n* = 1). In two patients, point mutations were found simultaneously in two genes: *WT1* and *BRCA2*, *TRIM28* and *CHEK2*. MLPA testing revealed large deletions of the 11p13 chromosomal region affecting the *WT1* gene in 4% of cases (*n* = 5), while epigenetic events in the 11p15 region were discovered in four cases (Figure 2).

#### 2.2.1. Genetic Alterations in the *WT1* Gene

**Figure 3:**
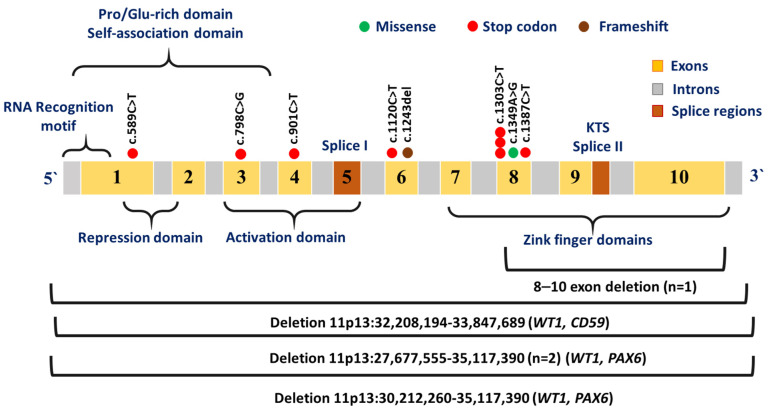
Localization of identified point mutations and large deletions in the *WT1* gene.

A complete list of genetic variations in the *WT1* locus is presented in Table 2 and Table S1. The vast majority of mutations in the *WT1* gene were associated with protein truncation and the consequent elimination of zinc finger domains encoded by exons 8–10 (eight nonsense mutations and one frameshift mutation, which also led to the appearance of a stop codon). Also, a missense mutation was identified in exon 8, which encodes one of the zinc finger domains (Figure 3).

In one patient, in addition to a mutation in the *WT1* gene, a pathogenic variant *BRCA2* c.8606_8607del (p.Ile2869fs) was identified (Table 2). Examination of the tumor tissue revealed loss of heterozygosity only for the *WT1* gene. The frequency of the variant allele of the *BRCA2* gene in the tumor was 30%. One patient had a deletion of exons 8–10 of the *WT1* gene; in three cases, the deletions affected the 11p13 chromosome region containing the *PAX6* and *WT1* genes, and in one case, it involved the *WT1* and *CD59* genes (Figure 3).

Seven of 15 patients (46%) had bilateral tumors; the most common histological subtype was stromal (13 of 22 affected kidneys, 59%). Various malformations were identified in 13 patients (86%), with urogenital abnormalities being the most common (8/15 or 53%), including bilateral pyelectasis, cryptorchidism, malformed labia, hypospadias, multiple renal masses, and anomalies of the contralateral kidneys. Disorders of sexual development (DSD) were observed in 5/6 male patients (83%) with *WT1* mutations. Skeletal anomalies (acrocephaly, congenital dysgenesis of the sacrum, elongation of the coccyx, clinodactyly, spina bifida) were noted in 3 cases (20%), neurological disorders (hydrocephalus, bilateral sensorineural hearing loss, delayed speech development) were also observed in 3 cases (20%), and heart defects in 2 cases (13.3%). Three patients with *PAX6* and *WT1* gene deletions had aniridia combined with WT, leading to a diagnosis of WAGR syndrome. The median age at diagnosis in the group of patients with *WT1* gene alterations was 13.3 months (range 3.9–27 months). In 6 (40%) patients, the tumor was diagnosed at stage I. Thirteen patients were alive at the last check-up, whilst two patients had died.

#### 2.2.2. Genetic Alterations in Other Genes

Patients with mutations in other genes, namely *TRIM28*, *REST*, *CHEK2*, *BRCA2*, *NF1*, *CDC73*, and *RAD50*, are presented in Table 3 and Table S1. Mutations in the *TRIM28* gene were loss-of-function variants (three frameshift mutations and one splice site mutation). All patients with *TRIM28* mutations had bilateral tumors, and the epithelial histological subtype was found in 7/8 affected kidneys (88%). Patients had various malformations, including urogenital anomalies in 50% of cases, and neurological problems (microcephaly, speech or motor development delay) in 50%, as well as facial dysmorphism, strabismus, and heart defects. One patient with a *TRIM28* mutation was found to have a second mutation, c.1100delC (p.Thr367fs) in the *CHEK2* gene.

Genetic variants in the *REST* gene were frameshift mutations in the DNA-binding domain, suggesting loss of protein function. One of the three patients (33%) had severe neurological disorders (autism, speech delay), as well as a femoral exostosis; another had grade 2 obesity and bilateral retinal angiopathy. A patient with the c.2668_2671del (p.Glu891Profs*6) mutation had bilateral WT and gingival hyperplasia, as described previously [25].

Pathogenic or likely pathogenic variants in the *NF1*, *CDC73*, *BRCA2*, and *RAD50* genes were identified, one in each patient. A patient with bilateral regressive nephroblastoma had a pathogenic variant c.5624dupA (p.Asn1875fs) in the *NF1* gene. The patient showed signs characteristic of neurofibromatosis type 1 (NF1): multiple café-au-lait spots and left-sided scoliosis.

A pathogenic variant c.1A>G (p.Met1Val) in the *CDC73* gene was identified in a one-year-old patient with bilateral WT of blastemal subtype, which developed against the background of diffuse hyperplastic perilobar nephroblastomatosis. The age at diagnosis was 8.8 months. The patient was born at 32 weeks of gestation and had multiple congenital malformations, including perinatal CNS damage, West syndrome, a congenital heart defect, a thoracic vein anomaly, affective-respiratory attacks, and bronchopulmonary dysplasia of prematurity.

A heterozygous nonsense mutation c.5286T>G (p.Tyr1762*) in the *BRCA2* gene was found in a patient with polycystic kidney disease and a lipoma. A genetic variant c.1928_1931del (p.Asp643Glyfs*30) in the *RAD50* gene leading to a frameshift and premature stop codon was classified as likely pathogenic according to the American College of Medical Genetics and Genomics (ACMG) criteria. The patient had a normal phenotype and received preoperative chemotherapy, but died due to a severe thrombotic complication of the underlying disease. Isolated mutations in the *CHEK2* gene were two splice site variants; one patient had a normal phenotype, while another was diagnosed with spina bifida.

The median age at WT manifestation in this group was 31.1 months (range 4.3–79.6 months), and in seven patients (53%), the tumor was diagnosed at stage I.

#### 2.2.3. Epigenetic Testing

Patients were referred for epigenetic testing if no pathogenic variants in cancer-associated genes or *WT1* rearrangements were found by multigene panel testing and MLPA analysis, and if characteristic phenotypic features of BWS/BWSp were presented. Bilateral WT was detected in 5 of 9 patients (56%), macroglossia in one (11%), lateralized overgrowth or hemihypertrophy in three (33%), hepatosplenomegaly in two (22%), pre- and postnatal macrosomia (birth weight > 2 SDS above the mean) in two (22%), and nevus flammeus on the head in one patient (11%). Other phenotypic manifestations included renal abnormalities, epicanthus, various facial abnormalities (micrognathia, prognathism, protruding ears, prominent forehead, and a flat nasal bridge). All patients were alive at the time of the last follow-up. The mean BWS score was determined to be 3.1 ± 0.7 according to established criteria (Table 4) [26]. In patients with a BWS score ≥ 4, a clinical diagnosis of BWS may be assigned (ID126, 127, 134).

MS-MLPA was carried out on both imprinting control regions 1 (IC1) and 2 (IC2) of chromosomal locus 11p15 using peripheral blood, tumor and healthy kidney samples (if applicable); the results are presented in Table 4. First-line molecular testing was performed using blood leukocyte DNA samples, and in three cases, a change in the 11p15 methylation pattern was detected (ID 130, 133, 134). In two of three (66%) patients, gain of methylation at IC1 (IC1 GOM) was found, and in one of three (33%), loss of methylation at IC2 (IC2 LOM) was identified. In patient ID128, IC1 GOM was found in DNA from healthy kidney tissue, suggesting a mosaic form of BWS. Aberrant methylation of the 11p15 locus was detected in tissue samples from the affected kidney in eight patients, while in patient ID134, this analysis was not possible due to complete necrosis of the affected kidney tissue. Finally, we confirmed the molecular diagnosis in 4/9 (44%) of patients suspected of having BWS.

### 2.3. Genotype-Phenotype Correlations

In total, 24% (32/134) of patients in our sample were found to have genetic or epigenetic aberrations. The median age of tumor manifestation in these patients was twice that of the median age in patients without aberrations (15.0 months, range from 2.5 to 79.6, vs. 30 months, range from 0 to 138 months). This difference is even more noticeable in patients with *WT1* aberrations, who had a median age at diagnosis of 13.3 months (range 3.9–27 months).

Bilateral kidney involvement was more common in patients with genetic or epigenetic aberrations compared to patients without aberrations (56% vs. 25%, OR = 3.8, 95% CI = 1.6–8.6, *p* = 0.004). The major contribution to the development of bilateral WT (70%) was observed for pathogenic variants in cancer-associated genes, other than *WT1*. The total genotype-phenotype associations identified in our study are presented in Table 5.

In general, patients with genetic and epigenetic aberrations were significantly more likely to have various abnormalities compared to patients without aberrations (86% vs. 25%, OR = 20.5, 95% CI = 6.6–63.9, *p* = 0.0008), and this was also observed for individual organ systems. Thus, patients with a mutant (epi)genotype compared to patients with wildtype (epi)genotype had more frequent anomalies of the reproductive (25% vs. 5%, OR = 6.5, 95% CI = 1.9–21.5, *p* = 0.005) and urinary (22% vs. 5%, OR = 5.4, 95% CI = 1.6–18.6, *p* = 0.01) systems, neurological impairment (22% vs. 2%, OR = 14.0, 95% CI = 2.7–71.6, *p* = 0.002), ophthalmological anomalies (16% vs. 3%, OR = 6.1, 95% CI = 1.4–27.2, *p* = 0.03), as well as other malformations (50% vs. 13%, OR = 6.8, 95% CI = 2.8–16.9, *p* = 0.0004). Anomalies of the reproductive and urinary systems were most often detected in patients with *WT1* gene aberrations (40% and 27%, respectively), while neurological and cognitive disorders were more common in patients with mutations in other cancer-associated genes (31%).

The main histological subtypes were evenly distributed among the group of patients without any aberrations, while in patients with an affected *WT1* gene, the stromal subtype predominated (64% vs. 14%, OR = 10.7, 95% CI = 3.4–29.6, *p* < 0.0001). In patients with mutations in the *TRIM28* gene, the epithelial subtype was much more common (88%).

**Figure 4:**
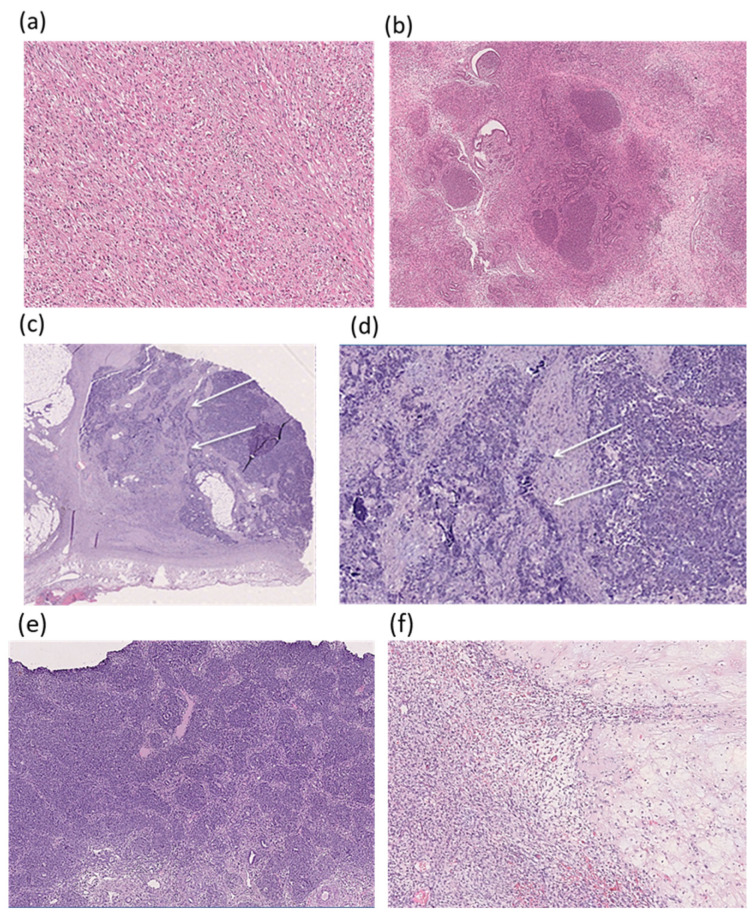
Histological subtypes of Wilms tumor in patients with different genotypes, stained with hematoxylin and eosin. (**a**)—Stromal type, mutation in the *WT1* gene (31 total cases, and 9 cases with *WT1* mutation). Spindle cell stroma with foci of maturation into rhabdomyoblasts, ×200. (**b**)—Mixed type, mutations in the *WT1* and *BRCA2* genes (26 total cases, and 4 cases with a mutation in the *WT1* gene). Tubular structures of the epithelial component with fields of undifferentiated blastema surrounded by spindle cell stroma, ×100. (**c**,**d**)—Focal anaplasia, mutation in the *WT1* gene (very rare variant was presented by one case): (**c**)—small (up to 1.5 cm) focus of anaplasia, separated from the rest of the tumor (indicated by arrows), ×100; (**d**)—large hyperchromic anaplastic nuclei, pathological mitoses are visible (indicated by arrows), ×200. (**e**)—Blastemal type, mutation in the *CDC73* gene (14 total cases). Fields of undifferentiated blastema with single tubular structures of the epithelium, ×100. (**f**)—Regressive type, mutation in the *NF1* gene (31 total cases). Focal necrosis at the border of the stromal component, in which single tubular epithelial structures are visible, ×200.

Examples of histological patterns in patients with mutations in various genes are shown in Figure 4. It should be noted that in patients with *WT1* alterations, in addition to the stromal subtype (Figure 4a), a mixed subtype (Figure 4b) and a rare variant with focal anaplasia were also revealed (Figure 4c,d). The patients with mutations in the *CDC73* and *NF1* genes had blastemal and regressive tumor types (Figure 4e and Figure 4f, respectively).

## 3. Discussion

Through our study, we confirm the significant proportion of hereditary forms in the etiology of Wilms tumor and provide important data on the spectrum and frequency of genetic abnormalities in a Russian patient cohort. The main result of our work is the identification of a genetic or epigenetic predisposition in 24% (*n* = 32) of patients with Wilms tumor, a rate exceeding the upper limit of the 10–15% range, which is indicated in several of the earliest studies [6,18,27,28]. This can be explained, on the one hand, by the in-depth genetic testing conducted, which not only included the search for point mutations using NGS, but also the analysis of chromosomal rearrangements (MLPA) and epigenetic abnormalities (MS-MPLA study) [15]. Such a comprehensive approach is the modern standard of diagnosis, since hereditary predisposition to Wilms tumor is extremely heterogeneous and is not limited to mutations in the *WT1* gene [9,29]. It is estimated that the prevalence of hereditary Wilms tumor may be even higher, reaching 33%, when investigating all currently discovered predisposing factors and not only using blood-derived DNA but also healthy kidney tissue DNA samples [7].

On the other hand, it should be noted that there is a high percentage of patients with bilateral WT (33%) in our sample, while in an unselected cohort of patients, the number of cases with two-sided kidney involvement usually does not exceed 5–10% [4,30]. In bilateral WT, like in familial forms, a high percentage of genetic and epigenetic aberrations has been noted [20,31]. In our study, the frequency of these alterations in bilateral WT was also significantly higher compared to unilateral tumors (21/44 or 48% vs. 16/90 or 18%, OR = 4.2, 95% CI = 1.9–9.4, *p* = 0.0004).

### 3.1. WT1 Gene Alterations

The *WT1* gene on chromosome 11p13 encodes a transcription factor that binds to gene promoters and enhancers, acting as an activator or repressor depending on the cellular context [1,8]. *WT1* gene alterations were the most common findings in our study and were detected in 11.2% (15 out of 134) of patients. The spectrum of these alterations was diverse: in addition to classic point mutations, we found large *WT1* deletions associated with both isolated forms of the disease and WAGR syndrome. An important observation was the predominance of the stromal histological tumor type among patients with *WT1* gene alterations, consistent with the known role of the *WT1* gene in the differentiation of mesenchymal precursors during the formation of renal tubules [8].

In our sample, frameshift mutations leading to protein truncation and loss of zinc finger domains predominated, in contrast to another study that reported a higher frequency of *WT1* missense mutations [29]. Among our patients, the missense mutation c.1349A>G (p.His450Arg) was found only once, and it is located in exon 8, which is marked as a hot spot in the *WT1* gene. This mutation, which results in the replacement of arginine with histidine in the penultimate codon of exon 8, has previously been described in a patient with Denys–Drash syndrome [32]. Our patient with the same mutation also presented with unilateral WT and a highly characteristic phenotype: bilateral pyeloectasis, cryptorchidism and early-onset disease at the age of 1 year. The most common mutation in the *WT1* gene in our sample, c.1303C>T (p.Arg435*), was observed in three patients (2.2%). The variant c.1243del (p.Met415Cysfs*39) has not been previously described in patients with WT, but its involvement in tumor development is highly probable, as this frameshift mutation leads to the loss of all zinc finger domains in the WT1 protein. Those with point mutations or deletions in the *WT1* gene had an early age of onset (median age 12 months), a high frequency of bilateral involvement (6/11 or 55%) and anomalies of the genitourinary system (6/11 or 55%), which are characteristic of *WT1*-associated diseases [33]. All three patients with *PAX6* and *WT1* gene deletions had aniridia, which, in combination with WT, is a manifestation of WAGR syndrome [34].

### 3.2. Impact of the TRIM28 and REST Genes

The genes *TRIM28* and *REST*, along with the *WT1* gene, are well-established predisposition genes for Wilms tumor. The TRIM28 protein (also known as KAP1/TIF1beta) is a transcription regulatory factor involved in various intracellular processes, including cell differentiation, DNA damage repair, apoptosis, and autophagy [35,36]. Mutations in the *TRIM28* gene were diagnosed in 3% of our patients, and in all cases, the tumor was of epithelial histological type and bilateral in location; also, the early age of tumor manifestation (12.5 months) was noteworthy. This corresponds to the recently described phenotype of *TRIM28*-associated hereditary nephroblastoma, which is characterized by this triad of features [19,36]. The mutation c.839+1G>A, which affects a donor splice site in intron 5 of the *TRIM28* gene, was previously identified in healthy kidney tissue and in tumors of WT patients [36,37]. We have shown that this variant may also be of germline origin. Three other *TRIM28* mutations revealed in our study (p.Glu388*, p.Cys669Profs*5, and p.Ser756Argfs*17) have not yet been described; all are truncating and therefore likely affect protein function. Given that loss of function is a known disease mechanism for *TRIM28*, these mutations may be considered high-risk drivers of Wilms tumor development. Moreover, no *TRIM28* mutations have been reported in patients with other childhood or adult cancers [18]. Thus, through our study, we further expand the spectrum of mutations in this gene.

The *REST* gene encodes a Kruppel-like transcription factor with a central DNA-binding domain composed of eight zinc fingers and two repressor domains and is involved in the regulation of transcription and chromatin remodeling [38,39]. In our study, *REST* mutations were detected in 3/134 (2.2%) cases, with bilateral tumors present in only one case (33%). In another study of patients with familial or bilateral Wilms tumors, the frequency of *REST* mutations was 4.7% [31]. It should be noted that all three of our patients had prominent phenotypic features: in one case, gingival fibromatosis and cerebral palsy; in a second, bilateral retinal angiopathy and obesity; and in a third, facial dimorphism and developmental delay. All three *REST* mutations are truncating. A case with c.2668_2671del (p.Glu891Profs*6) was described earlier in detail [25], and another variant c.831_832del (p.Cys278Trpfs*18) was reported in a familial Wilms tumor [39]. A novel mutation c.534del (Arg179Glufs*57) is localized in the zinc finger DNA-binding domain of the gene, where most pathogenic *REST* variants are primarily clustered [39]. This makes it highly likely that this mutation is also associated with Wilms tumor.

### 3.3. Genetic Variants in Other Genes

It is worth noting that pathogenic variants are identified in some patients in genes not typically associated with hereditary WT cases, such as *NF1*, *CDC73*, *BRCA2*, *CHEK2*, and *RAD50*.

In one case of bilateral WT, a pathogenic variant in the *NF1* gene was found in a patient who also had classic café-au-lait spots and was diagnosed with neurofibromatosis type 1. Although it is believed that the overall incidence of WT is significantly higher in patients with NF1 compared to the general population, this combination is extremely rare among all patients with WT [40,41,42]. The risk of Wilms tumor development in patients with NF1 is considered too low (>1%) to recommend WT surveillance for carriers of *NF1* mutations [9].

The *CDC73* gene encodes the protein parafibromin, a component of the PAF1 complex (PAF1C), which interacts with RNA polymerase II and regulates transcription elongation. Mutations in the *CDC73* gene are mainly associated with hereditary hyperparathyroidism-jaw tumor (HPT-JT) syndrome, parathyroid carcinoma, and kidney tumors, usually inherited in an autosomal dominant pattern [43]. Until recently, only a few patients with WT had been reported in unrelated families with HP-JT [44]. In our study, a c.1A>G (p.Met1Val) mutation was identified in a patient with bilateral WT, leading to a disruption of the initiation codon in mRNA. This variant has been described previously in individuals with *CDC73*-associated diseases [44,45], including WT [46]. The risk of developing WT for patients with *CDC73* mutations remains uncertain, and no surveillance is recommended in these cases [9].

Heterozygous pathogenic germline variants in adult-onset cancer predisposition genes were common findings in other childhood cancer studies [7,18,47]. However, biallelic mutations in the *BRCA2* gene have been described in patients with Fanconi anemia and familial Wilms tumor [48]. An extremely rare case of extrarenal Wilms tumor with a heterozygous mutation in the *BRCA2* gene in a 49-year-old woman has also been described [49]. In our patient with a heterozygous *BRCA2* mutation c.5286T>G, bilateral WT developed in the context of polycystic kidney disease. This pathogenic mutation has a population frequency of less than 0.0001% and was described earlier in Russian patients with breast cancer [50].

Mutations in the *CHEK2* and *RAD50* genes are considered moderate-risk variants of incomplete penetrance associated with multiple types of cancer. The *CHEK2* germline variants c.592+3A>T and c.444+1G>A with population frequencies 0.001% and 0.008%, respectively, have been described in multiple patients diagnosed with breast, prostate, thyroid, and stomach cancers [51,52,53]. The variant in the *RAD50* gene from our study was not found in healthy individuals from the Genome Aggregation Database (gnomAD).

In two of our cases, mutations in *CHEK2* and *BRCA2* were additional findings against a background of mutations in *WT1* and *TRIM28*; their role in predisposition to the development of tumor disease requires further study. Analyzing tumor DNA in a patient with mutations in two genes, *WT1* and *BRCA2*, showed that a loss of heterozygosity only occurred for the *WT1* mutation, suggesting that this event was the primary cause of the disease.

Although the heterozygous variants in adult-onset cancer genes are regularly detected in pediatric WT patients, their contribution to tumor development still remains unclear. The research in this area continues, and genes involved in repair processes and the maintenance of genome stability are of primary interest [54,55]. Even if mutations in these genes are unrelated to WT genesis, they may increase the risk of developing another cancer in children with WT in the future or influence sensitivity to anticancer therapy.

### 3.4. Aberrant Methylation of Locus 11p15

The overall percentage of molecularly confirmed BWS cases in our group was 3% (4/134), which corresponds to previously published reports [56,57]. Patients with BWS have an increased risk of developing Wilms tumor, especially those with IC1 GOM and pUPD11 [58,59,60], and even in non-syndromic patients, changes in the 11p15 region similar to those seen in BWS are frequently detected in tumor tissue [26]. In our study, epigenetic changes in tumor DNA were detected in nine patients with a BWS score ≥ 2, which is an indication for genetic testing of blood DNA. However, aberrant methylation patterns in blood-derived DNA were confirmed only in three patients, and in one case, a mosaic form of the syndrome was defined when healthy kidney-derived DNA was tested.

Additional investigation of resected healthy kidney tissue may significantly increase the diagnostic yield of BWS/BWSp [7]. Meanwhile, chromosome 11p15 aberrations detected in resected kidney tissue may represent tissue-specific somatic events [61]. Whether this patient group should be classified as constitutional mosaicism or a somatic phenomenon remains a matter of debate, especially if WT patients have at least one additional feature of BWSp. These patients could also be considered part of BWSp, suggesting that methylation changes in DNA obtained from blood may have been below the MS-MLPA detection threshold (approximately 10%). Furthermore, if the first-line MS-MLPA result is negative, it is recommended to search for mutations in the *CDKN1C* gene and for rare chromosomal abnormalities, or alternatively, to assign a diagnosis based only on clinical features [26]. In our study, two patients with a BWS score ≥ 4 could be classified as BWS without molecular confirmation.

The BWS phenotype in our cohort was quite variable, and the histological subtypes of tumors were diverse, which emphasizes the need for routine epigenetic testing in patients with WT, especially in those with macrosomia or other BWS stigmata, even in the absence of a classic clinical picture.

### 3.5. Clinical and Molecular Characteristics of Patients with WT

In our study, we revealed significant correlations between the presence of genetic or epigenetic aberrations and the clinical features of affected children. Patients diagnosed with CPS had a higher frequency of bilateral involvement (56%) and of various developmental abnormalities (86%). Due to the small number of participants in several groups compared and the too-wide confidence intervals, the associations may be considered as exploratory data requiring further verification in replication studies.

Meanwhile, some of these genotype-phenotype correlations were described previously [5,29,33,62,63]. Based on these studies, the McGill Interactive Pediatric OncoGenetic Guidelines (MiPOGG) algorithm was developed to rationalize referrals to genetic testing of children suspected of having a CPS. The algorithm includes five tumor-specific criteria (age of manifestation < 2 years, bilaterality or multifocality, stromal-predominant histology, nephrogenic rests, and overgrowth) and two universal criteria (family history suspicious for CPS and congenital anomalies) [28].

At the same time, the high genetic heterogeneity of Wilms tumor, the possible involvement of new or poorly studied genes, postzygotic mosaicism, and incomplete correlation between genotype and phenotype make it reasonable to conduct genetic testing in all children with Wilms tumor after consultation with a clinical geneticist [7,15]. In cases of limited possibilities, various algorithms (e.g., MiPOGG) can be used to determine the priority of patients referred to molecular genetic diagnostics. However, it should be noted that some cases with a CPS diagnosis may be missed; for example, in our study, two of 32 (6%) children with identified CPS did not meet the appropriate criteria for genetic testing, including one case with a mutation in the *WT1* gene.

In patients with identified CPS (*WT1*-associated tumor, Beckwith–Wiedemann syndrome, WAGR syndrome, etc.), the first-line therapy strategies can be immediately modified (application of nephron-sparing surgery and limited use of radiotherapy) with further implementation of individualized surveillance plans (ultrasound examination of the contralateral kidney, screening for secondary malignancies, e.g., hepatoblastoma in BWS) and genetic counseling of family members [9,28].

### 3.6. Limitations of the Study

The study has several limitations. First, the sample included a significant number of patients with bilateral tumors (33%) and various developmental anomalies (41%), who were referred to genetic consultation with suspected hereditary cancer syndromes. For this reason, the percentage of genetic and epigenetic aberrations in our study may be higher than in an unselected sample of patients with WT. Second, the NGS method is not designed to detect large deletions and chromosomal rearrangements. Additional MLPA testing was performed not for all patients without point mutations, but only for those having characteristic phenotypic features. Third, our study focused on investigating germline variants predisposing to Wilms tumor. At the same time, samples from the patients’ relatives were not always available to draw conclusions about the penetrance and pathogenicity of the genetic variants identified. Additional data on the pathogenesis of the disease and the role of genetic variants in tumor development could also be obtained by studying tumor DNA.

## 4. Materials and Methods

### 4.1. Patients

The study included 134 patients aged 0 to 20 years diagnosed with Wilms tumor during the observation period from March 2019 to February 2025 (6 years). Of these, 96 patients were treated at the N.N. Blokhin Russian Cancer Research Center of the Ministry of Health of the Russian Federation. These patients were referred from pediatric hospitals located in the European part of Russia (Central Russia, South Russia, Chuvashia and the North Caucasus). The remaining 38 patients were undergoing rehabilitation at the Rehabilitation Research Center “Russkoye Pole” of the Dmitry Rogachev National Medical Research Center of Pediatric Hematology, Oncology, and Immunology. These patients were referred from all regions of the Russian Federation, but mainly from Central Russia, the Moscow region and St. Petersburg. All patients from both Centers were White (approximately 70% of Slavic origin). For all patients, the following data were collected: histological findings, phenotype description, the presence of other tumors, and disease stage. Genetic testing was performed using NGS (130 samples), MLPA (8 samples), and MS-MLPA (9 samples). Genomic DNA was isolated from peripheral blood lymphocytes using the HiPure Blood DNA Mini Kit (Magen Biotechnology Co., Guangzhou, China) according to the manufacturer’s protocol. Tumor DNA was isolated from FFPE using the HiPure FFPE DNA Kit (Magen Biotechnology Co., Guangzhou, China) according to the manufacturer’s protocol. Before genetic testing, all patients signed an informed consent form.

### 4.2. Next-Generation Sequencing

For library preparation, a hybridization-based selective enrichment method was used with biotinylated probes targeting the coding regions of 415 genes associated with cancer development. The panel was designed using HyperDesign online service (Roche, Basel, Switzerland). The KAPA HyperPrep Kit (Roche, Basel, Switzerland) was used to prepare the libraries, and hybridization was performed using the KAPA HyperCap Workflow v3.0 kit (Roche, Basel, Switzerland). Sequencing was performed on a NextSeq2000 instrument (Illumina, San Diego, CA, USA) using the P1 300-cycle kit (Illumina, San Diego, CA, USA). The average coverage was 200–250×, the percentage of the target region with at least 50× coverage was ≥90%, and the quality score was ≥99.9%.

### 4.3. Bioinformatic Analysis

Sequencing quality was assessed using the FastQC tool. Read pairs were aligned to the reference genome (hg38) using the BWA–MEM2 algorithm. Then, duplicate sequences were identified and removed using the Picard MarkDuplicates program. Next, base quality scores were recalibrated, and genetic variants were identified using Genome Analysis Toolkit (GATK) tools: BQSR for recalibration of scores and HaplotypeCaller for variant calling. Base coverage uniformity exceeded 98% for all samples.

All samples with an average coverage ≥ 50× were included in further analysis. Variant annotation was performed using OpenCRAVAT version 3.1.1 (https://run.opencravat.org, accessed on 20 December 2025) software. Finally, all variants of interest were analyzed using Integrative Genomic Viewer (IGV) [64]. Interpretation of the identified variants was carried out according to the ACMG–AMP guidelines [65]. Variants were classified as benign (B), likely benign (LB), variants of uncertain significance (VUS), likely pathogenic (LP), or pathogenic (P). The following resources were used: open-access databases ClinVar (https://www.ncbi.nlm.nih.gov/clinvar, accessed on 15 January 2026), Varsome version 13.14.3 (https://varsome.com, accessed on 16 January 2026), and Franklin Genoox (https://franklin.genoox.com, accessed on 4 February 2026); population databases (Exome Sequencing Project, 1000 Genomes Project, Exome Aggregation Consortium); and in silico algorithms (SIFT, PolyPhen2–HDIV, PolyPhen2–HVAR, MutationTaster, MetaSVM, SpliceAI); as well as analysis of mutation type and location and literature data. If a variant was classified as “pathogenic” or “likely pathogenic”, it was verified by Sanger sequencing. When data and clinical material were available, family history was investigated and segregation analysis was performed.

### 4.4. MLPA Analysis

The SALSA MLPA Probemix P380–B1 Wilms tumor (MRC Holland, Amsterdam, The Netherlands) was used for the analysis. The SALSA MLPA Probemix P380–B1 Wilms tumor contains 53 MLPA probes with amplification products between 127 and 500 nucleotides (nt). This includes seven probes for chromosomal arm 1p, five probes for 1q, three probes for 16p, six probes for 16q, and three probes for each of the genes *MYCN*, *FBXW7*, *WT1*, *TP53*, and *AMER1*. Additionally, seven flanking probes (for the 2p, 2q, 4q, 11p, 17p, Xp, and Xq arms) are included to facilitate the determination of the extent of deletions or duplications. Ten reference probes target relatively copy-number-stable regions in various cancer types, including Wilms tumor. This probemix also contains nine quality control fragments generating amplification products between 64 and 105 nt: four DNA Quantity fragments (Q-fragments), two DNA Denaturation fragments (D-fragments), one Benchmark fragment, and one chromosome X- and one chromosome Y-specific fragment.

Sample preparation was performed according to the manufacturer’s protocol (the full protocol is available online at https://www.mrcholland.com). Fragment length analysis was performed on a 3500 Applied Biosystems instrument (Thermo Fisher Scientific, Waltham, MA, USA). Standard Coffalyser.Net (https://www.mrcholland.com/) data analysis software was used to analyze the data obtained.

### 4.5. MS-MLPA Analysis

The SALSA MLPA Probe mix ME030 BWS/RSS (MRC Holland, Amsterdam, The Netherlands) was used to perform MS–MLPA. The SALSA MLPA Probe mix ME030 BWS/RSS is an assay for the detection of aberrant methylation of one or more sequences of the following differentially methylated regions (DMRs): KCNQ1OT1: TSS-DMR (also called IC2), H19/IGF2: IG-DMR (also called IC1) in the 11p15 chromosomal region associated with BWS and Russell-Silver syndrome (RSS). This probe mix also includes probes for the IGF2:alt-TSS-DMR in the 11p15 chromosomal region. Additionally, this assay can be used for the detection of aberrant methylation of one or more sequences of the MEST: alt-TSS-DMR and GRB10: alt-TSS-DMR on chromosome 7 associated with RSS. This probe mix can also be used to detect deletions/duplications in the aforementioned chromosomal regions. Sample preparation was performed according to the manufacturer’s protocol (the full protocol is available online at https://www.mrcholland.com). Fragment length analysis was performed on a 3500 Applied Biosystems instrument (Thermo Fisher Scientific, Waltham, MA, USA). Standard Coffalyser.Net (https://www.mrcholland.com/) data analysis software was used to analyze the data obtained.

### 4.6. Statistical Analysis

For examination of statistical hypotheses, 2 × 2 contingency tables and Fisher’s exact test were applied. Odds ratios (OR) for genotype–phenotype associations were determined, and the 95% confidence intervals (95% CI) were reported. Statistical analysis was performed using GraphPad InStat 3 (GraphPad Software Inc., La Jolla, CA, USA). A *p*-value < 0.05 was considered statistically significant. The False Discovery Rate (FDR) was used to adjust for multiple comparisons.

## 5. Conclusions

Our study demonstrates the significant contribution of genetic and epigenetic factors to the development of Wilms tumor. Comprehensive genetic testing, including NGS, MLPA, and methylation analysis, may be considered the standard of care for this group of patients. The identified links between specific genetic aberrations and tumor histological subtypes deepen our understanding of the molecular mechanisms of Wilms tumor oncogenesis. Timely molecular diagnosis not only helps to explain the etiology of the disease but also significantly influences treatment strategies, long-term surveillance, and family counseling.

## Figures and Tables

**Table 1:** Clinical characteristics of the patients.

Characteristics		Number	%
Sex (*n* = 134)			
	Male	62	47%
	Female	72	53%
Median age (range), years		7 (from 2 to 20)	
Median age of manifestation (range), months		24.9 (from 0 to 147)	
Tumor pathology (*n* = 134)			
	Unilateral	90 (90/134)	67%
	Bilateral	44 (44/134)	33%
Histological subtypes (*n* = 166 affected kidneys)			
	Stromal	31 (31/166)	18.6%
	Regressive	31 (31/166)	18.6%
	Mixed	26 (26/166)	15.6%
	Epithelial	25 (25/166)	15.0%
	Nephroblastomatosis (NBM)	18 (18/166)	10.8%
	Blastemal	15 (14/166)	9.0%
	Non-anaplastic	9 (9/166)	5.4%
	Complete necrosis	5 (5/166)	3.0%
	Diffuse anaplasia	4 (4/166)	2.4%
	Focal anaplasia	1 (1/166)	0.6%
	Fetal rhabdomyomatousnephroblastoma	1 (1/166)	0.6%
	Undetermined	12	-
Wilms tumor only		130 (130/134)	97%
Other malignancies		4 (4/134)	2.9%
Clinical outcome (*n* = 129), lost to follow-up (*n* = 5)			
	Alive	122 (122/129)	95%
	—Complete remission	111 (111/129)	86%
	—Relapse	6 (6/129)	4.7%
	—Remaining tumor	5 (5/129)	4.3%
	Dead	7 (7/129)	5%

**Table 2:** Clinical and genetic data of patients with *WT1* genetic alterations. M—male, F—female, WT—Wilms tumor, UL—unilateral tumor (1), BL—bilateral tumor (2), NBM—nephroblastomatosis, *—termination codon, Mis—missense mutation, Non—nonsense mutation, Fms—frameshift mutation, Sl—start loss, Spl—splice site, Del—deletion, ACMG—American College of Medical Genetics, P—pathogenic, LP—likely pathogenic.

ID	M/F, Age at WT (Month)	UL/BL	Histology	Stage	Status, Age (Year)	Gene, Variant, Aminoacid, dbSNP or ClinVar ID	Type	ClinVar/ ACMG	Phenotype
113	M, 11	2	Stromal	II	Alive, 2	*WT1*, c.589C>T p.Gln197* ClinVar ID 4634848	Non	P/LP	Bilateral cryptorchidism, epicanthus
99	F, 27	2	Mixed/ NBM	I	Dead, 7	*WT1*, c.798C>G p.Tyr266* rs2133073037	Non	P/P	Multiple hemangiomas, malformed labia, heart defect
104	M, 13	1	Mixed	I	Alive, 5	*WT1*, c.901C>T p.Gln301* rs547775568	Non	LP/LP	Bilateral cryptorchidism, multiple hemangiomas
						*BRCA2*, c.8606_8607del p.Ile2869fs rs2548550872	Fms	P/P	
46	F, 27	2	Mixed	V	Alive, 14	*WT1*, c.1120C>T p.Arg374* rs1423753702	Non	P/P	Acrocephaly, sacrum dysgenesis, coccyx elongation, renal cyst
59	F, 10	2	Stromal	I	Alive, 4	*WT1*, c.1243del p.Met415Cysfs*39	Fms	-/LP	Hydrocephalus, facial dysmorphism, forehead hemangioma, clinodactyly
27	F, 12	1	Focal anaplasia	II	Alive, 10	*WT1*, c.1303C>T p.Arg435* rs121907906	Non	P/P	Congenital heart defects
49	F, 4	1	Stromal	I	Dead, 3		Non	P/P	Normal
103	M, 12	2	Stromal	V	Alive, 2		Non	P/P	Cryptorchidism, hypospadias, spina bifida, multiple renal masses
13	M, 12	1	Without anaplasia	I	Alive, 2	*WT1*, c.1349A>G p.His450Arg rs1851851609	Mis	LP/LP	Bilateral pyelectasis, cryptorchidism
68	F, 22	1	Without anaplasia	IV	Alive, 5	*WT1*, c.1387C>T p.Arg463* rs121907909	Non	P/P	Normal
121	F, 10	2	Stromal	I	Alive, 5	*WT1*, deletion 8–10 exons	Del	P	Bilateral pyelectasis
118	F, 23	1	Stromal	I	Alive, 5	*WT1*, *PAX6* deletion	Del	P	Convergent strabismus, aniridia, delayed speech development (WAGR)
119	M, 15	1	Stromal	I	Alive, 4	*WT1*, *PAX6* deletion	Del	P	Aniridia, macular hypoplasia, myopia, astigmatism, hypospadias, cryptorchidism, inguinal hernia (WAGR)
120	F, 14	1	Stromal	II	Alive, 4	*WT1*, *PAX6* deletion	Del	P	Congenital aniridia of both eyes (WAGR)
125	M, 19	2	Stromal	II	Alive, 5	*WT1*, *CD59* deletion	Del	P	Bilateral sensorineural hearing loss, additional lobe of the lung

**Table 3:** Clinical and genetic data of patients with genetic alterations. M—male, F—female, WT—Wilms tumor, UL—unilateral tumor (1), BL—bilateral tumor (2), NBM—nephroblastomatosis, *—termination codon, Mis—missense mutation, Non—nonsense mutation, Fms—frameshift mutation, Sl—start loss, Spl—splice site, Del—deletion, ACMG—American College of Medical Genetics, P—pathogenic, LP—likely pathogenic. Note: Genetic variants that are not currently included in the dbSNP may be added to the dbSNP repository by the authors at a later date.

ID	M/F, Age at WT (Month)	UL/BL	Histology	Stage	Status, Age (Year)	Gene	Variant, Aminoacid, dbSNP or ClinVar ID	Type	ClinVar/ ACMG	Phenotype
105	F, 6	2	Epithelial	I	Alive, 5	*TRIM28*	c.839+1G>A ClinVar ID 3655749	Spl	LP/P	Facial dysmorphism, genital anomalies
						*CHEK2*	c.1100delC Thr367Metfs*15	Fms	P/P	
19	M, 13	2	Epithelial	I	Alive, 3	*TRIM28*	c.2004_2005del p.Cys669Profs*5	Fms	-/LP	Congenital heart defects
65	M, 30	2	NBM/ Epithelial	I	Alive, 6	*TRIM28*	c.2268_2277del p.Ser756Argfs*17	Fms	-/LP	Neurologic impairment, strabismus
82	M, 41	2	Epithelial	IV	Dead, 8	*TRIM28*	c.1162G>T p.Glu388*	Non	-/LP	Cognitive impairment, genital anomalies
11	M, 80	1	Complete necrosis	I	Alive, 8	*REST*	c.534del Arg179Glufs*57	Fms	-/LP	Bilateral retinal angiopathy, grade 2 obesity
72	M, 25	1	Blastemal	II	Alive, 20	*REST*	c.831_832del Cys278Trpfs*18 rs869025310	Fms	P/P	Facial dysmorphism, cognitive impairment
101	F, 44	2	Regressive/ Blastemal	I	Alive, 8	*REST*	c.2668_2671del p.Glu891Profs*6	Fms	P/P	Gingival fibromatosis, cerebral palsy
74	F, 58	2	Regressive/ NBM	V	Alive, 13	*NF1*	c.5624dup p.Asn1875fs ClinVar ID 4713153	Fms	P/P	Multiple café-au-lait spots, scoliosis
5	M, 9	2	Blastemal	III	Alive, 2	*CDC73*	c.1A>G p.Met1Val rs1558276054	Sl	P/P	Multiple congenital malformations
50	F, 4	2	Mixed/ND	I	Alive, 3	*BRCA2*	c.5286T>G p.Tyr1762* rs80358754	Non	P/P	Polycystic kidney disease, axillary lipoma
20	F, 39	1	No operation	ND	Dead, 5	*RAD50*	c.1928_1931del p.Asp643Glyfs*30	Fms	-/LP	Normal
1	M, 32	1	Regressive	I	Alive, 5	*CHEK2*	c.592+3A>T rs587782849	Spl	P/LP	Spina bifida
71	F, ND	2	NBM	V	Alive, 11	*CHEK2*	c.444+1G>A rs121908698	Spl	P/P	Normal

**Table 4:** Epigenetic aberrations in patients with Wilms tumor suspected for BWS. M—male, F—female, UL—unilateral, BL—bilateral, N/A—not applicable, GOM—gain of methylation, LOM—loss of methylation.

ID	Sex, Age at WT (Month)	Age (Year)	UL/BL	Histology	Stage	Tissue			Phenotype	BWS Score
						Blood	Tumor	Healthy Kidney		
126	M, 56	7	2	Epithelial/ Regressive	V	No	IC1 (H19) GOM	No	Funnel chest, lateralized overgrowth	4
127	M, 42	6	2	Epithelial/ Necrotic	V	No	IC1 (H19) GOM	No	Lateralized overgrowth	4
131	F, 112	11	1	Regressive	I	No	IC1 (H19) GOM	No	Lateralized overgrowth	3
132	M, 49	8	2	Mixed/ NBM	II	No	IC1 (H19) GOM	No	Macrosomia	3
128	F, 18	6	2	Regressive/ NBM	I	No	IC1 (H19) GOM		Intraepidermal nevus on the left feet	2
129	F, 50	8	1	Epithelial	I	No	IC1 (H19) GOM	No	Hypotrophy, epicanthus, micrognathia, dyslalia	2
130	M, 12	5	1	Stromal	I	IC1 (H19) GOM		N/A	Facial dysmorphism, renal malformations, hepatosplenomegaly, macrosomia	3
133	M, 2	3	2	Epithelial/ NBM	V	IC1 (H19) GOM		N/A	Bilateral pyelectasis, cyst of kidney, hepatomegaly	3
134	F, 15	6	1	Complete necrosis	II	IC2 (CDKN1C) LOM	N/A	N/A	Macroglossia, facial dysmorphism, nevus flammeus on the head	4

**Table 5:** Comparison of clinical data in patients with and without aberrations; a statistically significant *p*-value (<0.05) is marked by (*). FDR-adjusted *p*-values are in brackets. (Epi)genetic—genetic and epigenetic, (epi)genotype—genotype and epigenotype, WT—Wilms tumor, mut—mutation, del—deletion, NBM—nephroblastomatosis, OR—odds ratio, CI—confidence interval, FDR—False Discovery Rate.

Clinics	(Epi)genetic Aberrations			Mutant (epi)genotype (n = 32), n (%)	Wildtype (epi)genotype (n = 102), n (%)
	WT1 mut/del (n = 15), n (%)	Other Genes (n = 13), n (%)	Epigenetics (n = 4), n (%)		
Laterality of WT					
Unilateral	8 (53%)	4 (30%)	2 (50%)	14 (44%)	76 (75%)
Bilateral	7 (47%)	9 (70%)	2 (50%)	18 (56%)	26 (25%)
Mutant 56% vs. wildtype 25%, OR = 3.8, 95% CI = 1.6–8.6, *p* = 0.002 * (*p* = 0.004 *)					
Genital abnormalities					
Present	6 (40%)	2 (15.3%)	0 (0%)	8 (25%)	5 (5%)
None	9 (60%)	11 (84.7%)	4 (100%)	24 (75%)	97 (95%)
Mutant 25% vs. wildtype 5%, OR = 6.5, 95% CI = 1.9–21.5, *p* = 0.003 * (*p* = 0.005 *)					
Kidney abnormalities					
Present	4 (27%)	1 (8.3%)	2 (50%)	7 (22%)	5 (5%)
None	11 (73%)	12 (91.7%)	2 (50%)	25 (78%)	97 (95%)
Mutant 22% vs. wildtype 5%, OR = 5.4, 95% CI = 1.6–18.6, *p* = 0.008 * (*p* = 0.01 *)					
Neurological or cognitive impairment					
Present	3 (20%)	4 (31%)	0 (0%)	7 (22%)	2 (2%)
None	12 (80%)	9 (69%)	4 (100%)	25 (78%)	100 (98%)
Mutant 22% vs. wildtype 2%, OR = 14.0, 95% CI = 2.7–71.6, *p* = 0.0006 * (*p* = 0.002 *)					
Ophthalmologic abnormalities					
Present	3 (25%)	2 (15%)	0 (0%)	5 (16%)	3 (3%)
None	12 (75%)	11 (75%)	4 (100%)	27 (84%)	99 (97%)
Mutant 16% vs. wildtype 3%, OR = 6.1, 95% CI = 1.4–27.2, *p* = 0.02 * (*p* = 0.03 *)					
Other malformations (cardiovascular, skin, skeletal anomalies, facial dysmorphism, macrosomia)					
Present	6 (40%)	7 (54%)	3 (100%)	16 (50%)	13 (13%)
None	9 (60%)	6 (46%)	1 (33%)	16 (50%)	89 (87%)
Mutant 50% vs. wildtype 13%, OR = 6.8, 95% CI = 2.8–16.9, *p* < 0.0001 * (*p* = 0.0004 *)					
Lateralized overgrowth					
Present	0 (0%)	0 (0%)	0 (0%)	0 (0%)	8 (8%)
None	15 (100%)	13 (100%)	4 (100%)	32 (100%)	94 (92%)
Mutant 0% vs. wildtype 8%, OR = 0.17, 95% CI = 0.01–3.1, *p* = 0.2 (*p* = 0.2)					
Total abnormalities					
Present	13 (87%)	11 (85%)	4 (100%)	28 (86%)	26 (25%)
None	2 (13%)	2 (15%)	0 (0%)	4 (14%)	76 (75%)
Mutant 86% vs. wildtype 25%, OR = 20.5, 95% CI = 6.6–63.9, *p* < 0.0001 * (*p* = 0.0008 *)					
Syndromes					
WAGR	3	0	0	3	0
BWS	0	0	4	3	0
Histological subtypes					
Affected kidney	*n* = 22	*n* = 21	*n* = 6		*n* = 117
Stromal	14 (64%)	0	1	-	16 (14%)
*WT1*-mutant 64% vs. wildtype 14%, OR = 10.7, 95% CI = 3.4–29.6, *p* < 0.0001 *					
Regressive	0	5 (24%)	1	-	25 (21%)
Mixed	4 (18%)	1 (5%)	0	-	21 (18%)
Epithelial	0	7 (33%)	1	-	17 (15%)
Blastemal	0	3 (14%)	0	-	12 (10%)
NBM	1 (4.5%)	3 (14%)	2	-	12 (10%)
Others	3 (13.5%)	2 (10%)	1	-	14 (12%)

## Data Availability

The data presented in this study are available within the article and Supplementary Materials; further inquiries can be directed to the corresponding author.

## Supplementary Materials

The following supporting information can be downloaded at: https://www.mdpi.com/article/10.3390/ijms27094066/s1.

## Abbreviations

The following abbreviations are used in this manuscript:

MLPA	multiplex ligation–dependent probe amplification
MS-MLPA	methylation-specific multiplex ligation–dependent probe amplification
BWS	Beckwith–Wiedemann syndrome
WAGR	Wilms tumor, aniridia, genitourinary anomalies, and range of developmental delays
